# Immune cell profiles of idiopathic inflammatory myopathy patients expressed anti-aminoacyl tRNA synthetase or anti-melanoma differentiation-associated gene 5 autoantibodies

**DOI:** 10.1186/s12865-023-00569-w

**Published:** 2023-09-26

**Authors:** Joung-Liang Lan, Shih-Hsin Chang, Gregory J. Tsay, Der-Yuan Chen, Yu-Hua Chao, Ju-Pi Li

**Affiliations:** 1 Rheumatology and Immunology Center, China Medical University Hospital, and School of Medicine, China Medical University, Taichung, Taiwan; 2 School of Medicine, Chung Shan Medical University, and Department of Pediatrics, Chung Shan Medical University Hospital, No.110, Sec.1, Jianguo N. Rd, Taichung City, 40201 Taiwan

**Keywords:** Idiopathic inflammatory myositis (IIM), Myositis-specific autoantibody (MSA), Aminoacyl-tRNA synthetase (ARS), Melanoma differentiation-associated gene 5 (MDA5), Immune cell profiles

## Abstract

**Background:**

Patients with idiopathic inflammatory myopathy (IIM) often express a different type of myositis-specific autoantibodies (MSAs), each associated with different clinical symptoms. Understanding the immunopathogenesis of various IIM subgroups can help improve the diagnosis and prognosis of IIM patients with different MSAs. However, the immune cell profiles of these IIM patients with anti-aminoacyl tRNA synthetase (ARS) or anti-melanoma differentiation-associated gene 5 (MDA5) autoantibodies remain unclear. We focused on the immune cell profiles of IIM patients with anti-ARS or anti-MDA5 autoantibodies.

**Results:**

The peripheral blood from IIM patients with anti-MDA5 autoantibody (MDA5 + group, *n* = 24) or one of the anti-ARS autoantibodies (ARS + group, *n* = 40) autoantibodies, and healthy controls (HC group, *n* = 60) were collected and examined. We found that IIM patients had a lower CD3 T cell population compared to the HC group. IIM patients showed a significantly lower T_N_ cell population and a higher T_EMRA_ cell population. Higher Th17 and Treg cell populations were found in these IIM patients than in the HC group. In these IIM patients, the MDA5 + group exhibited the higher percentages of Th17 and Treg cells than the ARS + group. It is noteworthy that the percentage of Th1 cells in the survival subgroup was higher than in the death subgroup in IIM patients with ARS + or MDA5 + . Furthermore, in the MDA5 + group, the percentage of Treg cells was higher in the survival subgroup compared to the death subgroup.

**Conclusions:**

Our study demonstrated that elevated Th1 may be a good prognostic indicator in IIM patients with ARS + or MDA5 + . Elevated Treg may also help predict a good prognosis in MDA5 + IIM patients. However, more large-scale studies and clinical samples are needed to verify the significance of Th1 and Treg cell subsets in clinical outcomes for these IIM patients with ARS + or MDA5 + . These data may help design a therapeutic approach that specifically targets the pathogenic immune molecular responsible for autoimmune attacks in IIM.

**Supplementary Information:**

The online version contains supplementary material available at 10.1186/s12865-023-00569-w.

## Background

Patients with idiopathic inflammatory myopathy (IIM), a rare autoimmune disease, generally show proximal muscle weakness, muscle inflammation, myalgia, fatigue, and various extramuscular manifestations [1, 2]. IIM patients often have one of various myositis-specific autoantibodies (MSAs), such as anti-aminoacyl tRNA synthetases (ARS) and anti-melanoma differentiation-associated gene 5 (MDA5) autoantibodies [3, 4]. Previous studies have shown that IIM patients with a certain type of MSA often have unique clinical manifestations [5, 6]. Therefore, the identification of MSAs is now considered to help identify various IIM subgroups [7, 8]. The most commonly detected MSAs in IIM patients are anti-ARS autoantibodies, including autoantibodies against histidyl- (Jo-1), alanyl- (PL-12), threonyl- (PL-7), asparaginyl- (KS), glycyl- (EJ), phenylalanyl- (ZO), tyrosyl- (HA), and isoleucyl- (OJ) tRNA synthetases [9, 10]. IIM patients with one of these anti-ARS autoantibodies often have typical clinical symptoms, known as antisynthetase syndrome, such as fever, myositis, inflammatory arthritis, mechanic's hands, Raynaud's phenomenon, and interstitial lung disease (ILD) [11–13]. While IIM patients with anti-MDA5 autoantibody often exhibit cutaneous features with muscle weakness (classical dermatomyositis) or without (clinical amyopathic dermatomyositis) and develop fatal rapidly progressive ILD, especially in Asian patients [14–16]. The presence of MSA indicates that an abnormal immune system is involved in the pathogenesis of IIM. Understanding the immunopathogenesis of various IIM subgroups can help in the design of optimal therapeutic strategies for IIM patients with different MSAs.

A dysregulated immune system is one of the leading causes of autoimmune diseases. The innate and adaptive immune systems are believed to be involved in the development of IIM [17, 18]. Recent studies have found that IIM patients had a reduction in the circulating subsets of CXCR3 + T cells and memory B cells [19–21]. Using immunocytochemical analysis, CD4 + and CD8 + T cells are found to mainly infiltrate the endomysial and perivascular regions of muscle tissues from polymyositis patients and the perivascular region of muscle tissues from dermatomyositis patients [22, 23]. Using mass cytometry by time-of-flight analysis (CyTOF), Th1, CXCR3 + Th2, CXCR3 + Th17 cells, and the CXCR3 + subsets of CD8 + cells are significantly reduced in IIM compared to healthy controls [19]. Using flow cytometry analysis, the immune phenotypes of anti-synthetase syndrome are shown to skew towards a Th1 phenotype [24]. Single-cell profiling data further demonstrate that interferon-stimulating gene + (ISG +) B cells, peripheral antibody-secreting cell clusters, and ISG15 + CD8 + T cell clusters are more enriched in patients with active MDA5 + dermatomyositis [25]. Although an abnormal T cell-mediated immune system has been shown to predominate in the pathogenesis of IIM, the role of T cells in the immunopathology of IIM with distinct MSAs is still under investigation. Our preliminary data showed that IIM patients with different autoantibodies exhibit unique peripheral blood T cell subsets [26]. In the present study, more participants were enrolled to examine immune cell profiles in the peripheral blood and to assess the associations between these immune cell profiles and the clinical outcomes.

## Results

### Clinical characteristics of study participants

To classify the different MSAs in IIM patients, a total of 268 IIM patients were enrolled. In this study, IIM patients with one of the anti-ARS autoantibodies (ARS + group, *n* = 40) and IIM patients with anti-MDA5 autoantibodies (MDA5 + group, *n* = 24) were selected to be further examined (Supplementary Fig. 1). Among these IIM patients with ARS + , there were Jo-1 (*n* = 13), EJ (*n* = 10), PL-12 (*n* = 7), OJ (*n* = 5), PL-7 (*n* = 3), and KS (*n* = 2). Furthermore, healthy participants were also enrolled as healthy control (HC group, *n* = 60). As shown in Table 1, the demographic characteristics of these participants were presented. The age of the participants in the ARS + group and the MDA5 + group was older than that of the HC group (Table 1). However, there were no significant differences in age and gender between the ARS + group and the MDA5 + group (Table 1). As Table 1 showed, the ARS + group and the MDA5 + group differed significantly in some clinical characteristics, including Gottron's sign (*p* < 0.01), skin rash (*p* = 0.02), and Raynaud's phenomenon (*p* = 0.03). Other clinical characteristics, such as periungual erythema, mechanic’s hand, muscle weakness, arthritis, and ILD, were similar between the groups (Table 1). Since some samples were collected on a different date than the date of blood test, laboratory data were only compared for 25 samples in the ARS + group and 18 samples in the MDA5 + group (Table 1). We observed that the ARS + group had a higher white blood cell (WBC) count compared to the MDA5 + group (*p* = 0.047, Table 1). No significant differences were found in neutrophil numbers, lymphocyte numbers, and neutrophil-to-lymphocyte ratio (Table 1). Furthermore, we checked that the samples were obtained before participants received immunosuppression. As shown in Table 1, 16 patients in the ARS + group (40.0%) and 8 patients in the MDA5 + group (33.3%) received immunosuppressive treatment prior to sample collection. Among these patients, more patients in the MDA5 + group received methotrexate compared with the ARS + group (*p* = 0.02, Table 1). There were no significant differences in the number of people using other immunosuppressive drugs (Table 1). Two patients lost follow-up in the MDA5 + group. Other participants in this study continued to receive the appropriate treatment for their condition. Table 1 showed that 5 out of 22 patients (22.7%) in the MDA5 + group and 1 out of 40 patients (2.5%) in the ARS + group were dead during the follow-up time. Therefore, a higher death rate was found in the MDA5 + group compared to the ARS + group (*p* < 0.01, Table 1).

**Table 1:** Demographics of patients with idiopathic inflammatory myopathy (IIM) and healthy control (HC)

Characteristics	IIM, ARS + (*n* = 40)	*IIM, MDA5* + (*n* = 24)	*P*^*1*^	*HC* (*n* = 60)	*p*^*2*^
Gender, n (%)					
Female	31 (77.5)	19 (79.2)	0.14	48 (80)	0.96
Male	9 (22.5)	5 (4.2)		12 (20)	
Age (years), median (IQR)	58.0 (47.0–67.0)	49.5 (43.4–58.8)	0.07	37.0 (33.0–43.3)	** < 0.01**
Organ involvement, n (%)					
Gottron's sign	10 (25.0)	15 (62.5)	** < 0.01**	NA	
Skin rash	13 (32.5)	15 (62.5)	**0.02**	NA	
Raynaud's phenomenon	15 (37.5)	3 (12.5)	**0.03**	NA	
Periungual erythema	14 (35.0)	11 (45.8)	0.39	NA	
Mechanic’s hand	20 (50.0)	14 (58.3)	0.52	NA	
Muscle weakness	20 (50.0)	7 (29.2)	0.10	NA	
Arthritis	16 (40.0)	9 (37.5)	0.84	NA	
Interstitial lung disease	18 (45.0)	15 (62.5)	0.18	NA	
WBC count (× 10^3/μl), median (IQR)	10.0 (8.4–15.7)^a^	7.6 (5.6–11.3)^b^	**0.047**	NA	
Neutrophil numbers (%)	77.0 (68.8–85.6)^a^	77.4 (70.8–86.8)^b^	0.71	NA	
Lymphocyte numbers (%)	10.6 (7.2–21.2)^a^	15.0 (5.7–17.6)^b^	0.70	NA	
NLR	8 (3.3–11.8)^a^	5.1 (4.0–15.8)^b^	0.67	NA	
Immunosuppressants, n (%)	16 (40.0)	8 (33.3)	0.28	NA	
Azathioprine	5 (12.5)	1 (4.2)	0.27	NA	
Cyclophosphamide	6 (15.0)	0 (0)	NA	NA	
Cyclosporin	3 (7.5)	3 (12.5)	0.51	NA	
Methotrexate	2 (5.0)	6 (25)	**0.02**	NA	
Mycophenolate	3 (7.5)	1 (4.2)	0.59	NA	
Tacrolimus	1 (2.5)	0 (0)	NA	NA	
Prednisolone ≥ 20 mg	4 (10.0)	2 (8.3)	0.82	NA	
Outcome, n (%)					
Alive	39 (97.5)	17 (77.3)^c^	** < 0.01**	NA	
Death	1 (2.5)	5 (22.7)^c^		NA	

Data are presented as a median (interquartile range, IQR) or n (%). ^a^*n* = 25, because other samples (*n* = 15) were collected on a different date than the complete blood count date; ^b^*n* = 18, because other samples (*n* = 6) were collected on a different date than the complete blood count date; ^c^*n* = 22, because 2 patients were lost to follow-up

Statistics: *p*^1^ value, calculated with Mann Whitney test or Chi square analysis between the ARS + and MDA5 + groups; *p*^2^ value, calculated with Kruskal–Wallis test or Chi square analysis among the MDA5, ARS, and HC groups

*Abbreviations*: *IIM* Idiopathic inflammatory myopathy, *ARS + * Anti-aminoacyl-tRNA synthetase autoantibody positive, *MDA5 + * Anti-melanoma differentiation-associated gene 5 autoantibody positive, *HC* Healthy control, *IQR* Interquartile range, *WBC* White blood cell, *NLR* Neutrophil-to-lymphocyte ratio, *NA* Non-applicable

### Different immune cell profiles between IIM patients with ARS + or MDA5 + and the HC group

**Fig. 1:**
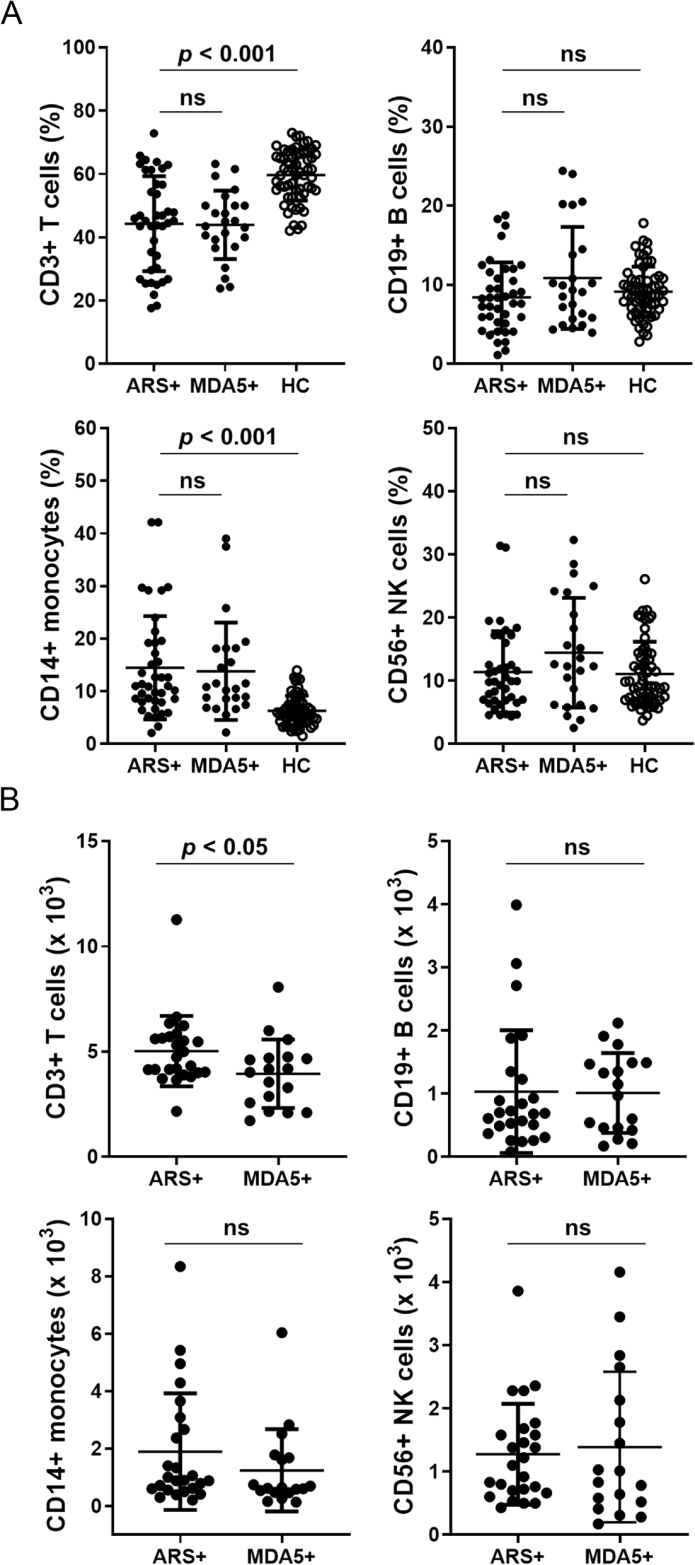
IIM patients have a lower CD3 + cell population and a higher CD14 + cell population in the pheripheral blood. **A** Flow cytometry analyses of CD3, CD14, CD19, and CD56 surface markers in peripheral blood mononuclear cells (PBMCs) of IIM patients with anti-aminoacyl-tRNA synthetase (ARS + , *n* = 40), or with anti-MDA5 antibody (MDA5 + , *n* = 24), and healthy controls (HC, *n* = 60). **B** Absolute cell numbers of CD3 + , CD14 + , CD19 + , and CD56 + cells of IIM patients with anti-aminoacyl-tRNA synthetase (ARS + , *n* = 25), or with anti-MDA5 antibody (MDA5 + , *n* = 18). Each dot was displayed for each data from the enrolled subjects. Data are presented as means ± SD. The *p* values were calculated using Kruskal–Wallis test between the ARS + , MDA5 + , and HC groups. ns, not significant

To evaluate specific immune profiles in IIM patients with ARS + or MDA5 + , the pheripheral blood of these IIM patients and HC was examined for CD3 + T cells, CD14 + monocytes, CD19 + B cells and CD56 + NK cell populations. Compared to the HC group, we found that the percentage of CD3 + T cells was lower (medium 45.1%, interquartile range (IQR) 34.5%-54.2% versus HC [60.8%, IQR 54.8%-66.4%]) (*p* < 0.001), while the percentage of CD14 + monocytes was higher (11.0%, IQR 8.0%-18.2% versus HC [5.7%, IQR 4.1%-7.8%]) (*p* < 0.001) in the peripheral blood of IIM patients (Fig. 1A). However, there were no differences in the percentages of CD19 + B cells and CD56 + NK cells between these IIM patients and the HC group (Fig. 1A). We further examine the immunophenotypes between these two IIM subgroups. No significant differences were observed in the percentages of CD3 + T cells, CD14 + monocytes, CD19 + B cells and CD56 + NK cells between the ARS + group and the MDA5 + group (Fig. 1A). Absolute lymphocyte numbers were obtained by combining WBC counts from the hematology analyzer (shown in Table 1) with flow cytometry population data. Compared to the MDA5 + group, the number of CD3 + T cells was higher in the ARS + group, but there were no differences in the number of CD14 + monocytes, CD19 + B cells, and CD56 + NK cells (Fig. 1B).

**Fig. 2:**
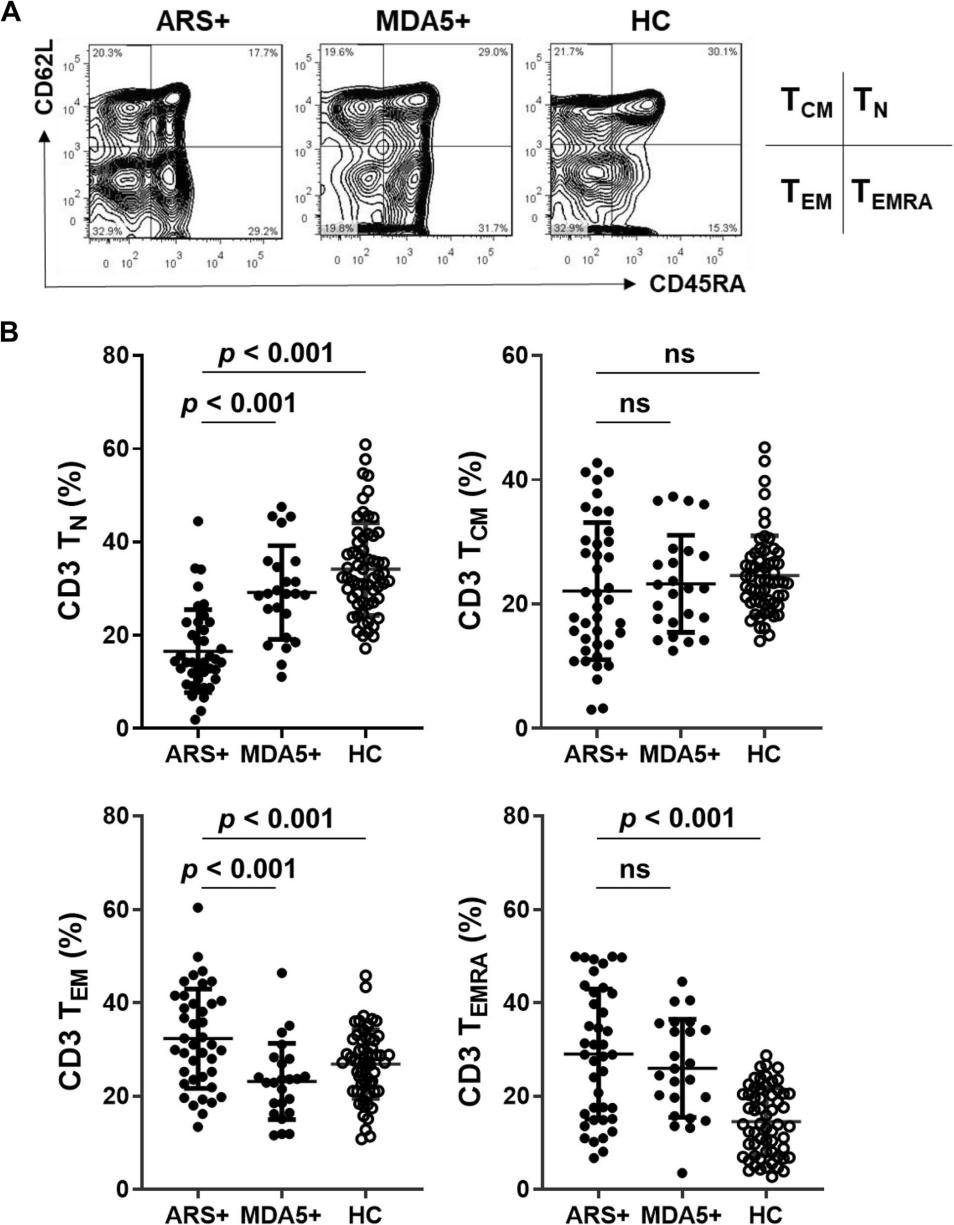
IIM patients exhibit a lower CD3 T_N_ cell population and a higher CD3 T_EMRA_ cell population in the pheripheral blood. Flow cytometry analyses of surface markers CD3, CD62L and CD45RA surface markers in PBMCs of IIM patients with anti-aminoacyl-tRNA synthetase (ARS + , *n* = 40), or with anti-MDA5 antibody (MDA5 + , *n* = 24), and healthy controls (HC, *n* = 60). **A** A representative data was shown. Naïve T (T_N_, CD45RA + CD62L +), central memory T (T_CM_, CD45RA-CD62L +), effector memory T (T_EM_, CD45RA-CD62L-), and terminally differentiated effector memory T cells (T_EMRA_, CD45R + CD62L-) (**B**) Each dot was displayed for each data from the enrolled subjects. Data are presented as means ± SD. The *p* values were calculated using Kruskal–Wallis test between ARS + , MDA5 + , and HC groups. ns, not significant

Since T cells have been shown to be involved in the pathogenesis of IIM [23], we examined T cell populations in IIM patients, the MDA5 + group, and the ARS + group. Compared to the HC group, IIM patients had a significantly lower CD3 cell population, including CD4 and CD8, in their pheripheral blood (*p* < 0.05, Table 2). Using surface expression of lymph node-homing markers, such as CD45RA and CD62L, T cells are subdivided into naïve cells (T_N_), central memory T cells (T_CM_), effector memory T cells (T_EM_) and terminally differentiated effector memory T cells (T_EMRA_) [27–31]. As shown in Fig. 2A, cell populations of T_N_ cells (CD45RA + CD62L +), T_CM_ (CD45RA-CD62L +), T_EM_ (CD45RA-CD62L-) and T_EMRA_ (CD45R + CD62L-) were examined. We observed a lower T_N_ cell population (*p* < 0.001) and higher T_EM_ and T_EMRA_ cell populations (*p* < 0.05, Fig. 2B and Table 2) in the pheripheral blood of IIM patients. However, the percentage of T_CM_ cells was similar between the IIM patients and the HC group (Fig. 2B and Table 2). Previous studies have shown that there are age-related changes in the distribution of pheripheral blood lymphocytes [32, 33]. Because our IIM patients were older than the HC group (Table 1), multiple linear regression analysis was performed to examine the effect of age, as a variant, on these T cell populations. After adjusting for age, these IIM patients still showed decreased T_N_ and increased T_EMRA_ cell population (*p* < 0.0001). However, after adjusting for age, there were no significant differences in T_EM_ (*p* = 0.92) and T_CM_ (*p* = 0.40) in these IIM patients. Furthermore, after adjusting for group (IIM), there was no significant difference in age (variant) for T_N_, T_EM_, T_CM_, and T_EMRA_ cell populations in the IIM and HC groups. We found that IIM patients with MDA5 (*n* = 24), Jo-1 (*n* = 13), EJ (*n* = 10), PL-12 (*n* = 7) and OJ (*n* = 5) showed a lower population of T_N_ cells compared to the HC group (Supplementary Fig. 2). IIM patients with MDA5 also showed a significantly lower population of T_EM_ cells compared to the HC group (Supplementary Fig. 2). Compared to the HC group, IIM patients with MDA5, Jo-1, and EJ, but not PL-12 and OJ, had a higher T_EMRA_ cell population (Supplementary Fig. 2). However, no significant differences were found in the percentage of T_CM_ cells in the MDA5 + group and the percentages of T_CM_ and T_EM_ cells in the ARS + group (Supplementary Fig. 2). Next, we compared these T cell subsets between the ARS + group and the MDA5 + group. The MDA5 + group had lower percentages of CD8 cells than the ARS + group (*p* < 0.01, Table 2). Compared to the MDA5 + group, the ARS + group showed a lower T_N_ cell population and a higher T_EM_ cell population in the subsets of CD3, CD4 and CD8 T cells (*p* < 0.05, Table 2 and Fig. 2B). Furthermore, no significant differences were found in the percentages of T_CM_ and T_EMRA_ cell populations in the subsets of CD3, CD4 and CD8 T cells between the two groups (Table 2 and Fig. 2B).

**Table 2:** Examination of T cell subsets in peripheral blood from patients with idiopathic inflammatory myopathy (IIM) and healthy control (HC)

Cell subsets	ARS + (*n* = 40)	*MDA5* + (*n* = 24)	*P*^*1*^	*HC* (*n* = 60)	*p*^*2*^
CD3 T (%)	51.0 (33.2–65.0)	43.4 (35.7–53.6)	0.22	64.5 (56.6–71.3)	** < 0.001**
CD3 T_N_ (%)	14.2 (10.6–22.3)	28.9 (20.8–35.5)	** < 0.01**	32.3 (27.0–40.7)	** < 0.001**
CD3 T_CM_ (%)	20.1 (13.5–30.2)	22.5 (17.2–28.3)	0.48	23.7 (20.2–27.4)	0.20
CD3 T_EM_ (%)	31.0 (23.7–40.3)	23.3 (16.9–27.7)	** < 0.01**	27.2 (21.5–32.1)	< **0.001**
CD3 T_EMRA_ (%)	28.9 (15.4–42.2)	25.2 (16.7–35.3)	0.46	14.0 (7.4–20.5)	** < 0.001**
CD4 T (%)	33.3 (22.5–40.5)	26.9 (21.0–29.9)	0.18	33.5 (27.6–40.0)	**0.02**
CD4 T_N_ (%)	16.7 (13.5–27.6)	27.3 (22.5–36.9)	** < 0.01**	41.1 (30.2–48.4)	** < 0.001**
CD4 T_CM_ (%)	25.0 (18.5–37.0)	28.2 (20.9–33.9)	0.60	29.2 (23.6–33.1)	0.85
CD4 T_EM_ (%)	31.4 (22.9–39.6)	20.1 (13.3–29.1)	** < 0.01**	21.3 (16.8–28.6)	** < 0.001**
CD4 T_EMRA_ (%)	16.6 (11.2–32.1)	18.9 (11.2–28.6)	0.77	8.8 (3.1–12.3)	** < 0.001**
CD8 T (%)	26.0 (15.4–32.6)	17.8 (10.6–20.5)	** < 0.01**	25.1 (20.5–29.1)	** < 0.001**
CD8 T_N_ (%)	17.1 (10.9–27.5)	29.4 (22.7–36.5)	** < 0.01**	42.5 (34.3–46.9)	** < 0.001**
CD8 T_CM_ (%)	8.8 (4.4–14.4)	7.7 (4.5–11.4)	0.82	9.1 (6.8–11.3)	0.36
CD8 T_EM_ (%)	28.9 (21.4–40.4)	24.0 (18.6–29.9)	**0.03**	29.7 (23.8–35.3)	**0.03**
CD8 T_EMRA_ (%)	35.6 (23.4–47.9)	40.2 (24.7–45.3)	0.60	18.2 (13.1–26.4)	** < 0.001**

Data are presented as a median (interquartile range, IQR)

Statistics: *p*^1^ value, calculated with Mann Whitney test between the MDA5 and ARS groups; *p*^2^ value, calculated with Kruskal–Wallis test among the MDA5, ARS, and HC groups

*Abbreviations*: *IIM* Idiopathic inflammatory myopathy, *ARS +*  Anti-aminoacyl-tRNA synthetase autoantibody positive, *MDA5 + * Anti-melanoma differentiation-associated gene 5 autoantibody positive, *HC* Healthy controls, *T*_*N*_ Naïve T cells, *T*_*CM*_ Central memory T cells, *T*_*EM*_ Effector memory T cells, *T*_*EMRA*_ Terminally differentiated effector memory T cells

### Higher populations of Th17 and Treg cells in peripheral blood of IIM patients with ARS + or MDA5 + , especially those with anti-MDA5 autoantibodies

**Fig. 3:**
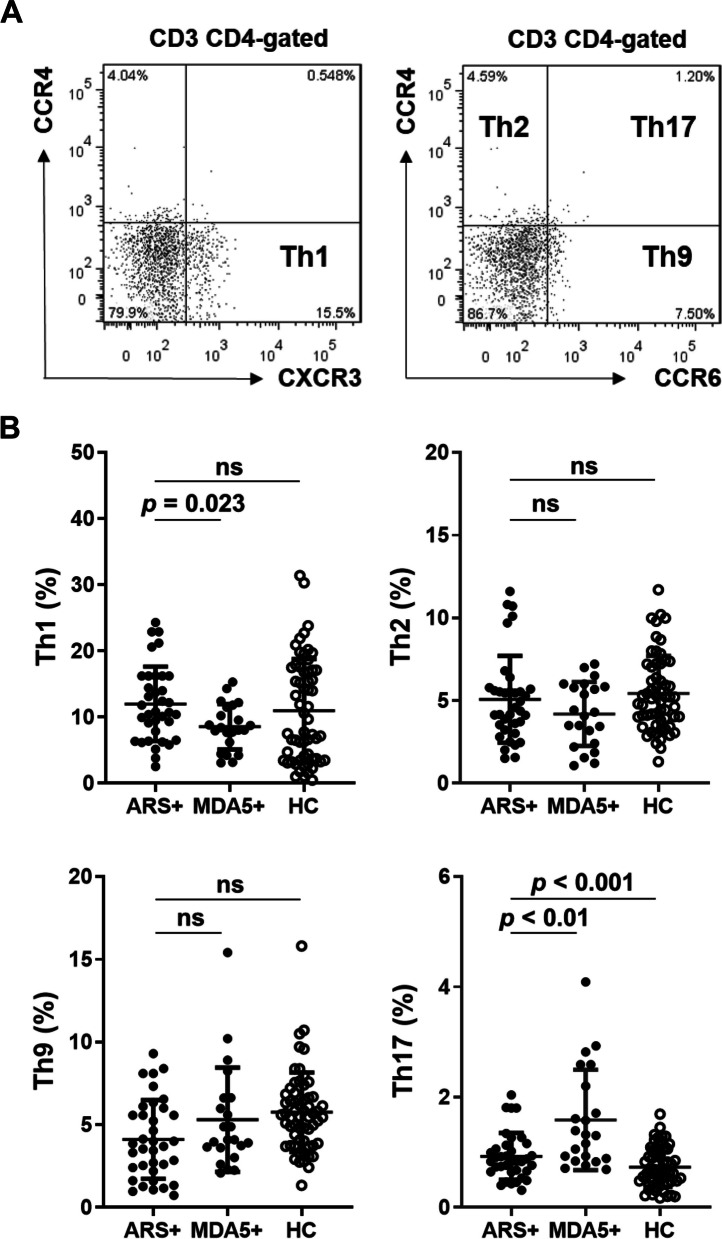
IIM patients with anti-MDA5 autoantibody have a higher Th17 cell population in the pheripheral blood. Flow cytometry analyses of CD3, CD4, CXCR3, CCR6, and CCR4 surface markers in PBMCs of IIM patients with anti-aminoacyl-tRNA synthetase (ARS + , *n* = 36), or with anti-MDA5 antibody (MDA5 + , *n* = 22), and healthy controls (HC, *n* = 60). **A** One represented data was shown. Th1, CXCR3 + CD4 + ; Th2, CXCR3-CCR4 + CCR6-CD4 + ; Th9, CXCR3-CCR4-CCR6 + CD4 + ; and Th17 cells, CXCR3-CCR4 + CCR6 + CD4 + . **B** Each dot was displayed for each data from the enrolled subjects. Data are presented as means ± SD. The *p* values were calculated using Kruskal–Wallis test between the MDA5, ARS, and HC groups. ns, not significant

The surface expression of the chemokine receptors, CXCR3, CCR6, and CCR4, is commonly used to classify the subsets of CD4 + T helper cells (Th), including type 1 (Th1), type 2 (Th2), type 9 (Th9) and type 17 (Th17) [34–37] (as shown in Fig. 3A). We found that IIM patients with ARS + or MDA5 + had significantly higher Th17 cell populations (1.0%, IQR 0.8%-1.5%) compared to the HC group (0.7%, IQR 0.5–1.1%) (*p* < 0.001; Fig. 3B). Compared to the HC group, IIM patients with OJ showed a higher Th1 cell population; with EJ or PL-12 had a lower Th9 cell population; with MDA5 (n = 22) had a higher Th17 cell population (Supplementary Fig. 3A). However, the percentage of Th1, Th2, and Th9 cells was not different between these IIM patients and the HC group (Fig. 3B). We performed Bonferroni's multiple comparison test to examine the differences between the ARS + and HC groups, the MDA5 + and HC groups, and the ARS + and MDA5 + groups. We found that the MDA5 + group also exhibited a higher percentage of the Th17 population (1.3%, IQR 0.9–2.3%) than the HC group and the ARS + group (0.8%, IQR 0.7–1.1%) (adjusted *p* < 0.01). The MDA5 + group showed a lower percentage of Th1 population (8.2%, IQR 5.8–11.9) than the ARS + group (10.8%, IQR 6.9%-16.2%) (*p* < 0.05), which was similar to the HC group (Fig. 3B). The ARS + group showed a lower percentage of Th9 population (3.8%, IQR 2.3–5.9) than the HC group (5.6%, IQR 3.9%-6.8%) (adjusted *p* < 0.01). Meanwhile, there were no significant differences in the percentage of the Th2 population between the groups (Fig. 3B).

**Fig. 4:**
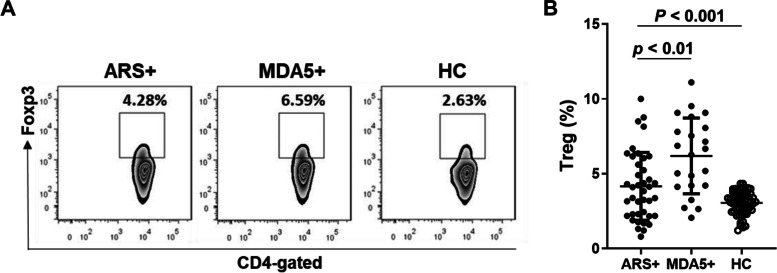
IIM patients with anti-MDA5 autoantibody have a higher Treg cell population in the pheripheral blood. Flow cytometry analyses of CD3, CD4, and Foxp3 markers in PBMCs of IIM patients with anti-aminoacyl-tRNA synthetase (ARS + , *n* = 40), or with anti-MDA5 antibody (MDA5 + , *n* = 22), and healthy controls (HC, *n* = 60). **A** A representative data was shown. Treg cells were defined as CD3 + CD4 + Foxp3 + . **B** Each dot was displayed for each data from the enrolled subjects. Data are presented as means ± SD. The *p* values were calculated using Mann–Whitney U test between the MDA5 and ARS groups, or using Kruskal–Wallis test between the MDA5, ARS, and HC groups

The subsets of regulatory T (Treg) cells suppress the activation and function of other leukocytes to maintain immune homeostasis [38, 39]. Furthermore, we examined the circulating Treg cell populations in these IIM patients with ARS + or MDA5 + and the HC group (Fig. 4A). As shown in Fig. 4B, the percentage of Treg cells in peripheral blood was significantly higher in IIM patients (4.3%, IQR 2.7–6.7%) compared to the HC group (3.1%, IQR 2.6–3.6%) (*p* < 0.001). Using Bonferroni's multiple comparison test, we found that IIM patients with ARS + or MDA5 + showed a higher Treg cell population compared to the HC group (adjusted *p* < 0.01). Additionally, IIM patients with OJ or MDA5 also showed a higher Treg cell population compared to the HC group (Supplementary Fig. 3B). The MDA5 + group also showed a higher percentage of the Treg population (6.5%, IQR 4.2–8.3%) than the ARS + group (4.0%, IQR 2.2–5.9%) (*p* < 0.01, Fig. 4B). Therefore, IIM patients with ARS + or MDA5 + , especially those with anti-MDA5 autoantibody, had a higher percentage of Treg cells compared to the HC group.

### A higher proportion of Treg cells is associated with survival in IIM patients with anti-MDA5 autoantibodies

**Fig. 5:**
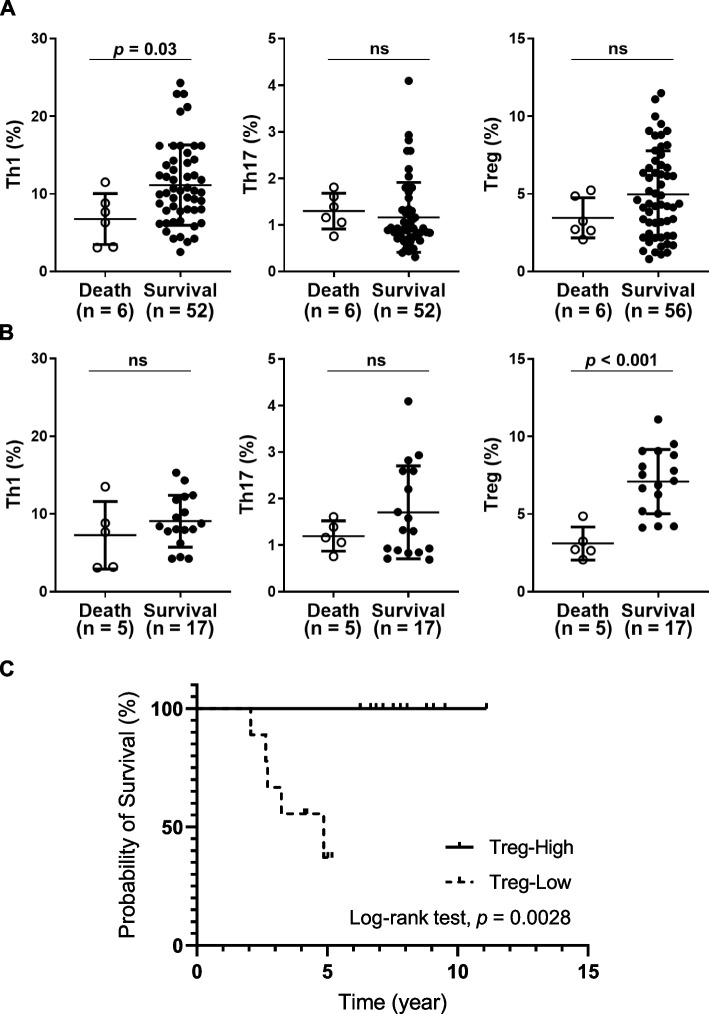
A higher Th1 cell population is associated with survival in IIM patients. **A** Differences in Th1, Th17, and Treg cell populations between death and survival in the IIM patients with ARS + and MDA5 + were compared. **B** Differences in Th1, Th17, and Treg cell populations were compared between death (*n* = 5) and survival (*n* = 17) in the MDA5 + group. Each dot was displayed for each data from the enrolled subjects. Data are presented as means ± SD. The *p* values were calculated using the Mann–Whitney U test. ns, not significant. **C** The cumulative incidence of overall survival in the MDA5 + group according to their Treg levels (*n* = 22). Treg-High, the Treg level was higher than average Treg level. Treg-Low, lower than average Treg level. The *p* values were calculated using log-rank test

The association between these immune cell profiles and patient death / survival was examined during the study period. We found that the percentage of Th1 cells was higher in the survival subgroup (10.3%, IQR 7.8–14.2%) compared to the death subgroup (7.0%, IQR 3.1–9.5%) in IIM patients with ARS + or MDA5 + (*p* < 0.05, Fig. 5A). However, there were no significant differences in the percentages of Th17 and Treg cells between the two groups in IIM patients with ARS + or MDA5 + (Fig. 5A). We observed that in the MDA5 + group, the percentage of Treg cells in the survival subgroup (7.1%, IQR 5.1–8.9%) was significantly higher than in the death subgroup (2.7%, IQR 2.4%-4.1%) (*p* < 0.001, Fig. 5B). To further distinguish the effect of Treg levels on the cumulative incidence of survival in the MDA5 + group, we set the mean 6.14% of the Treg percentage as the cut-off value. In the MDA5 + group, Treg-High referred to samples whose Treg level was higher than the cutoff value; conversely, Treg-Low referred to samples whose Treg value was lower than the cutoff value. The Kaplan–Meier survival plot showed a significant difference in the cumulative incidence of survival between Treg-High and Treg-Low (*n* = 22, Fig. 5C; *p* = 0.0028, log-rank test). That is, the cumulative incidence of overall survival was significantly higher in IIM patients with high Treg levels in the MDA5 + group. These data suggest that IIM patients with ARS + or MDA5 + with a higher Th1 cell population may have a better survival trend. IIM patients with anti-MDA5 autoantibodies with a larger population of Treg cells may have a better survival trend.

## Discussion

As the abnormal adaptive immune system is predicted to participate in IIM pathogenesis, various subsets of circulating T cells were examined in IIM patients with different MSAs. Here, we observed that IIM patients exhibited a lower T_N_ cell population and higher T_EM_ and T_EMRA_ cell populations, although IIM patients had a lower T cell population compared to the healthy control group. IIM patients with anti-ARS autoantibodies also showed a lower T_N_ cell population and a higher T_EM_ cell population than those with anti-MDA5 autoantibody. We found higher percentages of Th17 and Treg cells in peripheral blood of IIM patients with ARS + or MDA5 + , especially those with anti-MDA5 autoantibodies. In particular, the percentage of Th1 cells in the survival subgroup was higher than in the death subgroup in IIM patients with ARS + or MDA5 + . Furthermore, in the MDA5 + group, the percentage of Treg cells was higher in the survival subgroup compared to the death subgroup. Here we found that elevated Th1 may be a good prognostic indicator in IIM patients with ARS + or MDA5 + . Additionally, elevated Treg may also help predict a good prognosis in MDA5 + IIM patients.

IIM, a heterogeneous group of systemic autoimmune diseases, is characterized clinically by weakness of skeletal muscles and pathologically by muscle infiltration of leukocytes, preferentially T cells and macrophages [1, 2, 40–43]. Recently, IIM has been known to be classified into several clinical serological subgroups: polymyositis, dermatomyositis (DM; including adult and juvenile (JDM)), amyopathic DM, antisynthetase syndrome, and inclusion body myositis (IBM) [44]. In addition, immune-mediated necrotizing myopathy, hypomyopathic DM, cancer-associated myositis and overlap myositis are also clinically classified. Effector memory T cells can drive the pathogenesis of autoimmune diseases because they have ready-made effector functions for the presence of autoantigens [45]. Here, we observed that IIM patients with anti-MDA5 or anti-ARS autoantibodies (including Jo-1, EJ, PL-12, and OJ) showed lower populations of T_N_ cells compared to the HC group. Meanwhile, these IIM patients with MDA5, Jo-1, and EJ also had higher populations of T_EMRA_ cells compared to the HC group. These data suggest that peripheral blood T cells in IIM patients may differentiate toward effector memory T cells, resulting in a decreased T_N_ cells and the increased T_EMRA_ cells compared to healthy control subjects. Previous studies demonstrate that memory T cell differentiation is significantly increased in systemic lupus erythematosus [46] and rheumatoid arthritis [47]. Highly differentiated CD8 + T cell populations have been found in the peripheral blood of IBM patients, a subgroup of IIMs that frequently harbor anti-cytosolic 5'-nucleotidase 1A (cN1A) autoantibodies [48]. We found that IIM patients with one of anti-ARS autoantibodies had an increased T_EM_ cells compared to the MDA5 + group. The data indicate that circulating T cells may predominately differentiate into effector memory T cells, including CD4 + and CD8 + , leading to the immunopathogenesis of autoimmune diseases. Therefore, these different effector memory T cells may further contribute to the pathogenesis of IIM subsets.

In the present study, we found that IIM patients with anti-ARS autoantibody showed higher percentages of circulating Th1 cell populations than those with anti-MDA5 autoantibody. In fact, Nelke et al. found that immunophenotypes in peripheral blood samples from patients with anti-synthetase syndrome are mainly differentiated toward the Th1 phenotype [24]. We observed that IIM patients with anti-MDA5 autoantibody exhibited higher percentages of circulating Th17 cell populations than healthy controls. Previous studies have shown that Th17-associated pro-inflammatory cytokines must be detected in lymphocytic infiltrates of IIM muscles, indicating that Th17 cell subsets may contribute to chronic muscle inflammation in IIM patients [49, 50]. Consistently, the frequency of circulating Th17 cells increased significantly in IIM patients [51]. However, Wilfong et al. found that CXCR3 + Th17 cells were significantly decreased in IIM patients compared to healthy controls [19]. Ye et al. demonstrated that IIM patients with MDA5 + in active and remission stages have different states of the adaptive immune system [25]. Therefore, these controversial results require the careful study design. A previous study demonstrates that the amount of Treg increases in inflamed muscle tissues [52]. On the contrary, IBM patients had a lower percentage of Treg cells compared to healthy controls [53]. Our finding that IIM patients have the increased number of circulating Treg cells suggests that the immune system may still struggle to maintain immune homeostasis during IIM pathogenesis, especially with anti-MDA5 autoantibody. Since the observation period varied from case to case, using Kaplan–Meier survival plots and log-rank tests, we observed a significantly higher cumulative incidence of survival in the MDA5 + group with higher Treg levels. Therefore, monitoring Treg levels may be helpful for the diagnosis of the MDA5 + group. These data suggest that IIM patients with a unique autoantibody intrinsically exhibit the distinct T cell immune system.

There are several limitations in the present study. For example, older healthy controls (> 55 years) were not enrolled in the present study; although we used multiple linear regression analysis to surmise that age did not affect our data. However, we were unable to provide an accurate comparison of T cell populations between the HC and IIM groups. Our data provided the immune status of the T cells of each subject at the time of receipt, but may not be the initial status of the IIM. We found that several patients in this study received immunosuppressive therapy before sample collection. It cannot be ruled out that the use of these drugs may affect the levels of lymphocyte subsets in these IIM patients. Furthermore, the causes of death are often complex, including infection, drug side effects, and other factors that may alter the immune status; therefore, further studies are needed to evaluate patient samples during treatment and before/after different treatment regimens. More human subjects and multicenter studies are needed to assess and confirm these findings.

## Conclusion

Taken together, our data showed that higher percentages of Th17 and Treg cells in peripheral blood of IIM patients, especially those with anti-MDA5 autoantibodies. The percentage of Th1 cells in the survival subgroup was significantly higher than that in the death subgroup in IIM patients with ARS + or MDA5 + . In the MDA5 + group, the percentage of Treg cells was higher in the survival subgroup compared to the death subgroup. These data can further help improve the diagnosis and prognosis of IIM patients with anti-ARS or anti-MDA5 autoantibodies.

## Methods

### Study participants

IIM patients (*n* = 268) who met the 1975 criteria described by Bohan and Peter [54, 55] were consecutively enrolled in China Medical University Hospital from 2015 to 2020 (Supplementary Fig. 1). The following patients were included: (1) patients showed clinical manifestations of myositis; (2) patients had interstitial lung disease but no evidence of myositis (amyopathic group). The exclusion criteria were as followed: (1) patients met criteria for other connective tissue diseases; (2) the enrolled subjects were unable to take venous blood; (3) those IIM patients were younger than 20 years old and older than 80 years old. The clinical assessment of the patients at the time of serum sampling was based on a retrospective evaluation of patient records by a physician. Healthy participants without rheumatic diseases and related family histories were enrolled as healthy controls (HC, *n* = 60) (Supplementary Fig. 1). This study was approved by the Institutional Review Board of the China Medical University Hospital (CMUH104-REC3-093). The informed consent form was obtained from each participant according to the Declaration of Helsinki.

### Specimen preparation

Whole blood samples from all participants were collected at the time of the first visit. The sera were obtained by centrifugation at 1,200 rpm for 5 min and stored at -80 °C for the subsequent determination of MSAs. Peripheral blood mononuclear cells (PBMCs) were isolated by Ficoll-Paque density centrifugation (GE Healthcare Biosciences, Illinois, USA) and examined using flow cytometry analysis.

### MSAs determination

A total of 268 sera from IIM patients were examined using a line blot assay according to the manufacturer’s instructions (EUROLINE Autoimmune Inflammatory Myopathies 16 Ag test kit; Euroimmune, Germany). The reaction intensity (2 + or 3 +) determined by the manufacturer's scanning software was considered positive. To avoid false positives, several ELISA kits (MBL, Nagoya, Japan) were used to verify the presence of anti-ARS antibodies (including anti-Jo-1, anti-EJ, anti-PL-7, anti-PL-12, and anti-KS antibodies) and anti-MDA5 antibody.

### Flow cytometry analysis

PBMCs were harvested and stained with fluorochrome-conjugated antibodies panels [56]. To examine immune cell populations, the PBMCs were stained with PE-Cy7-conjugated anti-CD3 (HIT3a), PE-conjugated anti-CD19 (HIB19), PerCP-conjugated anti-CD14 (HCD14), and Alexa-fluor 488-conjugated anti-CD56 (HCD56). To explore the T cell compartments, the PBMCs were stained with PE-Cy7-conjugated anti-CD3 (HIT3a), PerCP-Cy5.5-conjugated anti-CD4 (RPA-T4), PE-conjugated anti-CD8 (RPA-T8), Alexa-fluor 647-conjugated anti-CD62L (DREG-56), and Alexa-fluor 488-conjugated anti-CD45RA (HI100). To classify T cell subsets, PBMCs were stained with PE-Cy7-conjugated anti-CD3 (HIT3a), PerCP-Cy5.5-conjugated anti-CD4 (RPA-T4), Alexa-fluor 647-conjugated CCR6 (11A9), Alexa-fluor 488-conjugated anti-CXCR3 (1C6), and PE-conjugated anti-CCR4 (1G1). To identify the Treg cell population, PBMCs were stained with PE-Cy7-conjugated anti-CD3 (HIT3a), PerCP-Cy5.5-conjugated anti-CD4 (RPA-T4), and Alexa-fluor 647-conjugated anti-CD25 (BC96) and fixed. After permeabilization with FOXP3 Fix/Perm Buffer Set (BioLegend, San Diego, CA, USA), these cells were stained with Alexa-fluor 488-conjugated anti-Foxp3 (259D/C7). These antibodies were obtained from BioLegend (USA) and BD Pharmingen (USA). Data were acquired with a FACSCelesta instrument (BD Biosciences, USA) and analyzed using FlowJo analytical software (TreeStar, USA).

### Statistical analysis

Categorical variables were shown as numbers with percentage (%), and continuous variables were presented as mean ± standard deviation (SD). Flow cytometry data were expressed as median with interquartile ranges (IQR). Statistical analyses, including Chi square analysis, Mann–Whitney test, and Kruskal–Wallis test, were performed to evaluate the significant differences between these groups. For survival analysis, Kaplan–Meier method and the log-rank test were performed. Data analyses were carried out with GraphPad Prism 7.0 software (USA). Two-sided *p*-values less than 0.05 were considered statistically significant.

### Supplementary Information

**Additional file 1:** **Supplementary Figure 1.** Flowchart of the analysis process. **Supplementary Figure 2.** Various T cell populations in the pheripheral blood of IIM patients with different autoantibodies. Flow cytometry analyses of surface markers CD3, CD62L and CD45RA surface markers in PBMCs of IIM patients with anti-Jo-1 (*n* = 13), EJ (*n* = 10), PL-12 (*n* = 7), OJ (*n* = 5), or MDA5 (*n* = 24) autoantibody and healthy controls (HC, *n* = 60). Naïve T (TN, CD45RA+CD62L+); central memory T (TCM, CD45RA-CD62L+); effector memory T (TEM, CD45RA-CD62L-); and terminally differentiated effector memory T (TEMRA, CD45R+CD62L-). Each dot was displayed for each data from the enrolled subjects. Data are presented as means ± SD. *, *p* < 0.05. The p values were calculated using the Mann-Whitney U test between each subgroup and the HC group. **Supplementary Figure 3.** Various T cell subsets in the pheripheral blood of IIMpatients with different autoantibodies. (A) Flow cytometry analyses of CD3, CD4, CXCR3, CCR6, and CCR4 surface markers in PBMCs of IIM patients with anti-Jo-1 (*n* = 9), EJ (*n* = 10), PL-12 (*n* = 7), OJ (*n* = 5), or MDA5 (*n* = 22) autoantibody and healthy controls (HC, *n* = 60). Th1, CXCR3+CD4+; Th2, CXCR3-CCR4+CCR6-CD4+; Th9, CXCR3-CCR4-CCR6+CD4+; and Th17 cells, CXCR3-CCR4+CCR6+CD4+. (B) Flow cytometry analyses of CD3, CD4, and Foxp3 markers in PBMCs of IIM patients with anti-Jo-1 (*n* = 9), EJ (*n* = 10), PL-12 (*n* = 7), OJ (*n* = 5), or MDA5 (*n* = 22) autoantibody and healthy controls (HC, *n* = 60). Treg cells were defined as CD3+CD4+ Foxp3+. Each dot was displayed for each data from the enrolled subjects. Data are presented as means ± SD. *, *p* < 0.05. The *p* values were calculated using the Mann-Whitney U test between each subgroup and the HC group.

## Data Availability

The datasets generated and/or analyzed during the current study are not publicly available due to privacy and ethical concerns but are available from the corresponding author on reasonable request.

## Abbreviations

IIM: Idiopathic inflammatory myopathy
MSA: Myositis-specific autoantibody
ARS: Aminoacyl tRNA synthetase
MDA5: Melanoma differentiation-associated gene 5
HC: Healthy control
ILD: Interstitial lung disease
IQR: Interquartile range
T_N_: Naïve cells
T_CM_: Central memory T cells
T_EM_: Effector memory T cells
T_EMRA_: Terminally differentiated effector memory T cells
Th: T helper cells
Treg: Regulatory T
DM: Dermatomyositis
IBM: Inclusion body myositis
WBC: White blood cell
NLR: Neutrophil-to-lymphocyte ratio
