# Supplementary material for: Immune cell profiles of idiopathic inflammatory myopathy patients expressed anti-aminoacyl tRNA synthetase or anti-melanoma differentiation-associated gene 5 autoantibodies
Source: BMC Immunol. 2023 Sep 26;24:33. doi: 10.1186/s12865-023-00569-w (PMC10523699; doi:10.1186/s12865-023-00569-w)

Immune Cell Profiles of Idiopathic Inflammatory Myopathy Patients Expressed Anti-Aminoacyl tRNA Synthetase or Anti-Melanoma Differentiation-Associated Gene 5 Autoantibodies

Joung-Liang Lan[1], Shih-Hsin Chang[1], Gregory J Tsay[1], Der-Yuan Chen[1], Yu-Hua Chao[2], Ju-Pi Li[2*]

Supplementary information

Supplementary Figure 1. Flowchart of the analysis process.

Supplementary Figure 2. Various T cell populations in the pheripheral blood of IIM patients with different autoantibodies. Flow cytometry analyses of surface markers CD3, CD62L and CD45RA surface markers in PBMCs of IIM patients with anti-Jo-1 (n = 13), EJ (n = 10), PL-12 (n = 7), OJ (n = 5), or MDA5 (n = 24) autoantibody and healthy controls (HC, n = 60). Naïve T (T_N, CD45RA+CD62L+); central memory T (T_{CM}, CD45RA-CD62L+); effector memory T (T_{EM}, CD45RA-CD62L-); and terminally differentiated effector memory T (T_{EMRA}, CD45R+CD62L-). Each dot was displayed for each data from the enrolled subjects. Data are presented as means ± SD. *, $p < 0.05$. The p values were calculated using the Mann-Whitney U test between each subgroup and the HC group.

Supplementary Figure 3. Various T cell subsets in the pheripheral blood of IIM

patients with different autoantibodies. (A) Flow cytometry analyses of CD3, CD4, CXCR3, CCR6, and CCR4 surface markers in PBMCs of IIM patients with anti-Jo-1 (n = 9), EJ (n = 10), PL-12 (n = 7), OJ (n = 5), or MDA5 (n = 22) autoantibody and healthy controls (HC, n = 60). Th1, CXCR3+CD4+; Th2, CXCR3-CCR4+CCR6-CD4+; Th9, CXCR3-CCR4-CCR6+CD4+; and Th17 cells, CXCR3-CCR4+CCR6+CD4+. (B) Flow cytometry analyses of CD3, CD4, and Foxp3 markers in PBMCs of IIM patients with anti-Jo-1 (n = 9), EJ (n = 10), PL-12 (n = 7), OJ (n = 5), or MDA5 (n = 22) autoantibody and healthy controls (HC, n = 60). Treg cells were defined as CD3+CD4+ Foxp3+. Each dot was displayed for each data from the enrolled subjects. Data are presented as means ± SD. *, $p < 0.05$. The p values were calculated using the Mann-Whitney U test between each subgroup and the HC group.

Supplementary Figure 1

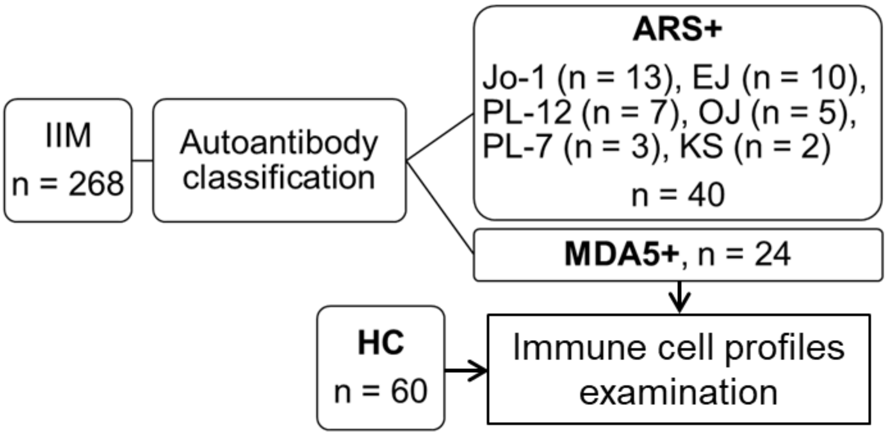

Supplementary Figure 2

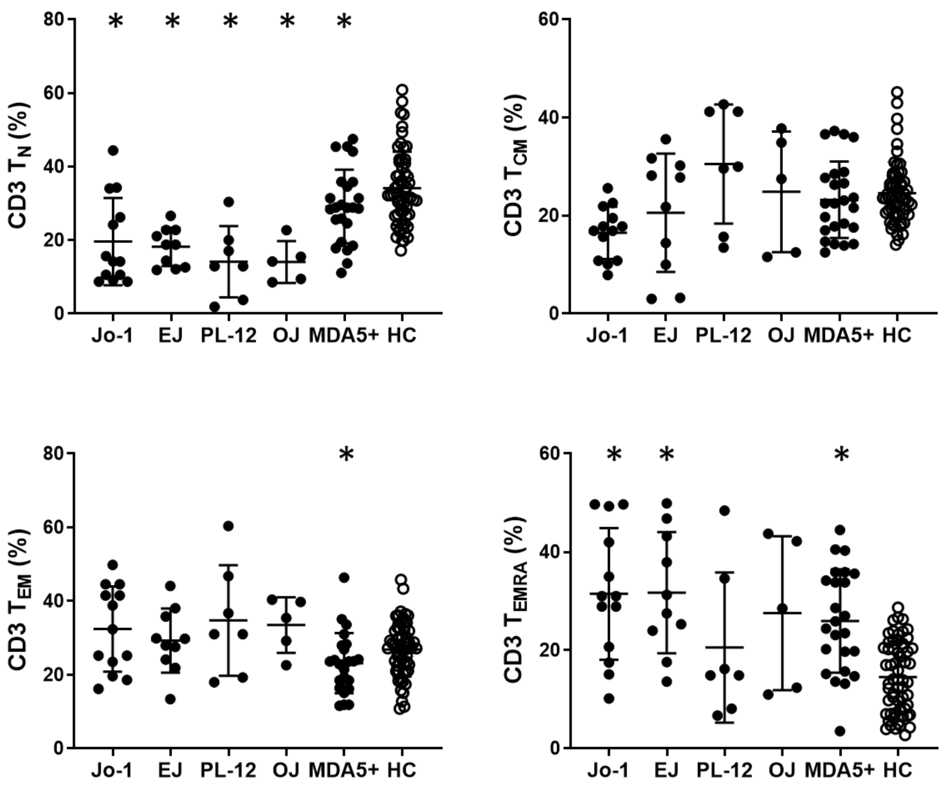

Supplementary Figure 3

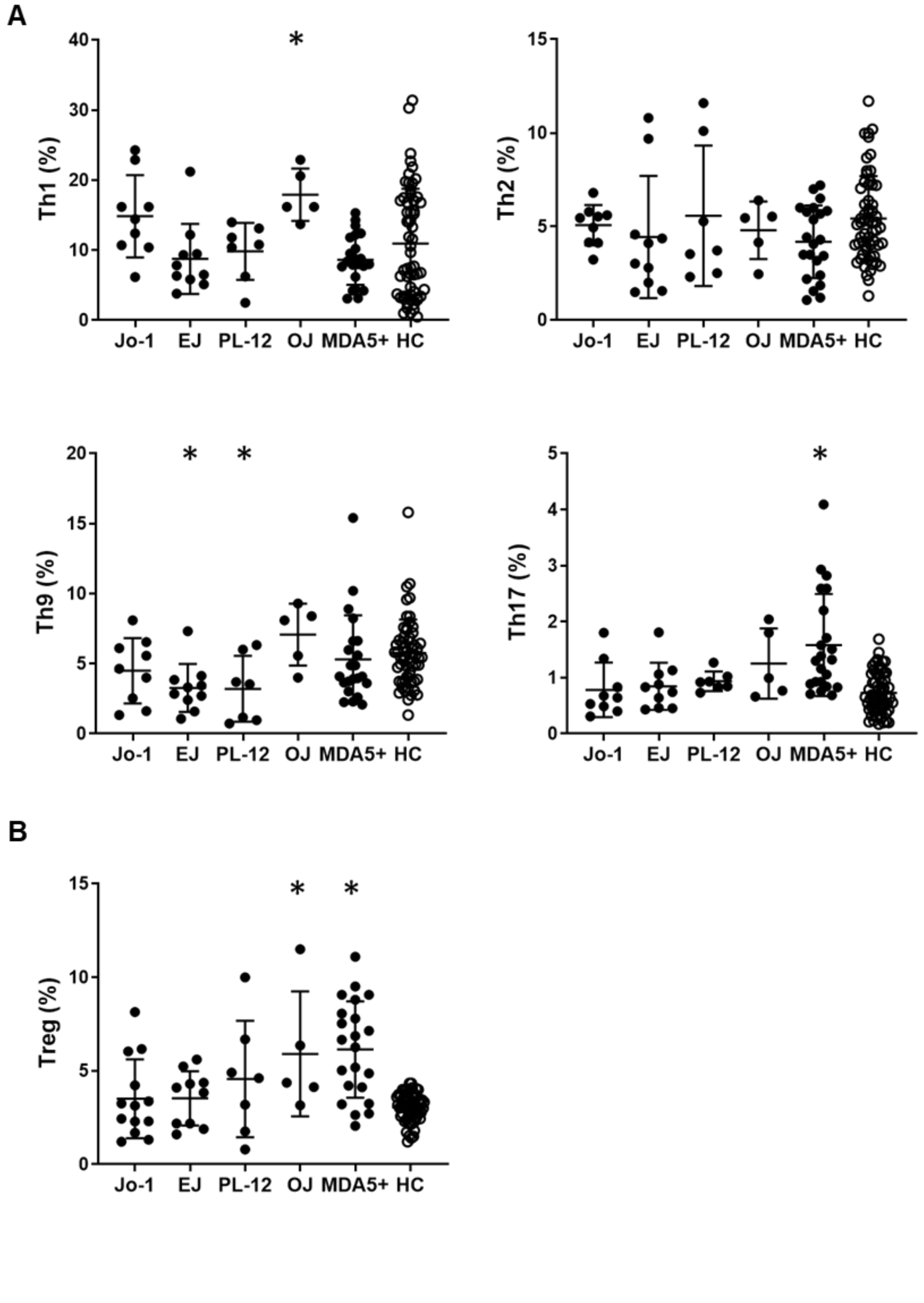

Supplement: Supplementary file 1 — Additional file 1: Supplementary Figure 1. Flowchart of the analysis process. Supplementary Figure 2. Various T cell populations in the pheripheral blood of IIM patients with different autoantibodies. Flow cytometry analyses of surface markers CD3, CD62L and CD45RA surface markers in PBMCs of IIM patients with anti-Jo-1 (n = 13), EJ (n = 10), PL-12 (n = 7), OJ (n = 5), or MDA5 (n = 24) autoantibody and healthy controls (HC, n = 60). Naïve T (TN, CD45RA+CD62L+); central memory T (TCM, CD45RA-CD62L+); effector memory T (TEM, CD45RA-CD62L-); and terminally differentiated effector memory T (TEMRA, CD45R+CD62L-). Each dot was displayed for each data from the enrolled subjects. Data are presented as means ± SD. *, p < 0.05. The p values were calculated using the Mann-Whitney U test between each subgroup and the HC group. Supplementary Figure 3. Various T cell subsets in the pheripheral blood of IIMpatients with different autoantibodies. (A) Flow cytometry analyses of CD3, CD4, CXCR3, CCR6, and CCR4 surface markers in PBMCs of IIM patients with anti-Jo-1 (n = 9), EJ (n = 10), PL-12 (n = 7), OJ (n = 5), or MDA5 (n = 22) autoantibody and healthy controls (HC, n = 60). Th1, CXCR3+CD4+; Th2, CXCR3-CCR4+CCR6-CD4+; Th9, CXCR3-CCR4-CCR6+CD4+; and Th17 cells, CXCR3-CCR4+CCR6+CD4+. (B) Flow cytometry analyses of CD3, CD4, and Foxp3 markers in PBMCs of IIM patients with anti-Jo-1 (n = 9), EJ (n = 10), PL-12 (n = 7), OJ (n = 5), or MDA5 (n = 22) autoantibody and healthy controls (HC, n = 60). Treg cells were defined as CD3+CD4+ Foxp3+. Each dot was displayed for each data from the enrolled subjects. Data are presented as means ± SD. *, p < 0.05. The p values were calculated using the Mann-Whitney U test between each subgroup and the HC group. [file 12865_2023_569_MOESM1_ESM.pdf]
